# Characterization of the complete chloroplast genome of *Brunfelsia brasiliensis* (Spreng.) L.B.Sm. & Downs

**DOI:** 10.1080/23802359.2025.2492096

**Published:** 2025-04-17

**Authors:** Shaoping Wu, Hong Nie, Jinyan Liao, Fei Wang, Xing Long, Junwen Huang

**Affiliations:** a School of Life Sciences, Zhaoqing University, Zhaoqing, China; b School of Computer Science and Software, Zhaoqing University, Guangdong, China; c Gaoyao No. 2 Middle School, Zhaoqing, China; d Horticultural Research Institute, Guangxi Academy of Agricultural Sciences, Nanning, China

**Keywords:** Chloroplast genome, *Brunfelsia brasiliensis*, phylogenetic analyses

## Abstract

*Brunfelsia brasiliensis* (*B. brasiliensis*) (Spreng.) L.B.Sm. & Downs is a perennial evergreen shrub that is widely cultivated as an ornamental plant in tropical and subtropical regions. This study presents the complete chloroplast genome of *B. brasiliensis*, having a length of 169,062 bp. The chloroplast genome contains 137 genes, including 92 protein-coding, 37 tRNA genes, and 8 rRNA genes. Phylogenetic analysis reveals that *Petunia hybrida* and *P. exserta* are closely linked to *B. brasiliensis* in evolutionary terms. This first chloroplast genome assembly is a valuable resource for future genetic and molecular biology studies of the genus *Brunfelsia.*

## 1. Introduction

*B. brasiliensis* is a perennial evergreen shrub from the *Solanaceae* family, classified under the *Brunfelsia* genus. Native to Brazil, this genus includes over 50 species and is widely cultivated as an ornamental plant in tropical regions. *B. brasiliensis* grows to 70–150 cm in height and is characterized by numerous branches and dark brown stems with vertical fissures in the bark. Its leaves are simple, alternately arranged, and long-lanceolate in shape. The flowers, primarily solitary with occasional clusters, feature a shallowly lobed, chalice-like corolla. They have an extended blooming period and exhibit a unique transition in color: the blooms change from deep blue-violet to soft lavender and eventually to white. This color change is driven by dynamic changes in the proportion of endogenous pigment molecules in the plants. This simultaneous display of different flower colors has led to its common name, ‘Yesterday-Today-and-Tomorrow’, in various languages. Due to its significant ornamental value, *B. brasiliensis* is widely cultivated in outdoor parks and used for the interior decoration of buildings throughout Mediterranean Europe, as well as the tropical and subtropical regions of Asia.

However, the limited genetic information on *B. brasiliensis* has left the molecular mechanisms responsible for changes in flower color largely unexplored. Additionally, chloroplasts, essential organelles responsible for pigment storage, play a crucial role in carbon assimilation during photosynthesis, supplying the necessary materials for synthesizing pigments and fragrances. This study assembled and annotated the first chloroplast genome for this important ornamental plant. This research may provide new insights into the color transition of the flower and the biosynthesis of its fragrance compounds in *B. brasiliensis*.

## 2. Materials and methods

**Figure 1.**
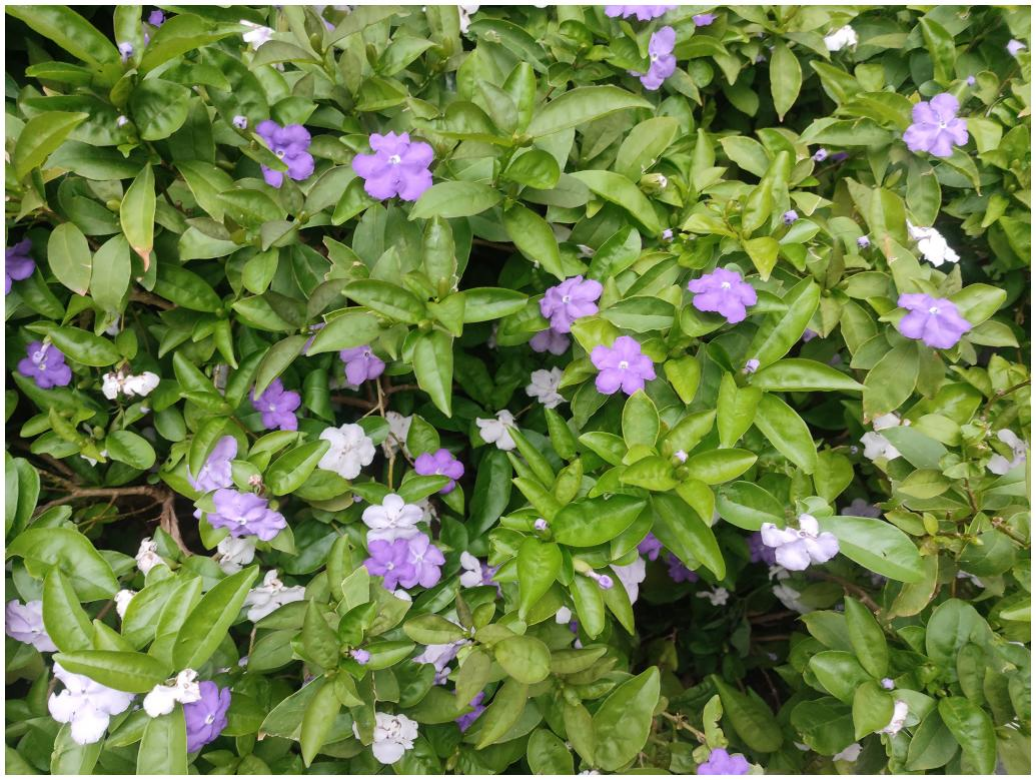
Plants of *B. brasiliensis* were collected from the plant germplasm reservation base of Zhaoqing University (captured by Dr. Junwen Huang). The stem is dark brown and exhibits strong branching capabilities. The individual leaves are alternate, papery, and have smooth, entire margins without teeth. The flowers are clustered in a funnel shape, forming inflorescences. The corolla has five lobes with serrated petals. During the bud stage, the flowers are mushroom-shaped and dark purple. The fragrance is rich, and the two-colored flowers bloom simultaneously on the branches. The leaves possess medicinal properties.

Samples of *B. brasiliensis* were collected from the plant germplasm preservation base of Zhaoqing University (23° 6′ 33″ N; 112° 29′ 38″ E) (Figure 1). The specimen of *B. brasiliensis* was deposited in the herbarium of Zhaoqing University (Contact: Dr. Junwen Huang, junwenH@yeah.net) under the voucher number *YYML-20240608*.

High-quality genomic deoxyribonucleic acid (DNA) of *B. brasiliensis* was extracted from the young leaves using the cetyltrimethylammonium bromide method (Doyle and Doyle 1987) following an optimized protocol. A DNA library was then constructed from the extracted total genomic DNA and evaluated using the Agilent 5400 system. Chloroplast genome sequencing was conducted on the Illumina HiSeq 4000 platform (Novogene). After sequencing, the raw data from Novogene was analyzed, resulting in several filtered reads, each with a mean length of 150.0 bp. The low-quality reads were removed using the GetOrganelle for chloroplast genome assembly (Supplementary Figure 1).

The annotation process proceeds using PGA https://github.com/quxiaojian/PGA)software (Qu et al. 2019), with the results integrated into Geneious Prime for manual refinement. The chloroplast genome primarily consists of protein-coding (CDS), tRNA, and rRNA genes. Each tRNA gene was calibrated using an online annotation tool, ARAGORN (Laslett and Canback 2004), while the physical map of the entire chloroplast was visualized in a circular format using an Organellar Genome DRAW online tool (Greiner et al. 2019). In the evolutionary analysis, the XSEDE system was utilized to generate a maximum likelihood (ML) phylogenetic tree (Supplementary Figure 2), with 1000 bootstrap replicates produced using RA x ML-HPC BlackBox (version 8.2.12) (https://www.phylo.org/portal2/home.action). The IQ-tree software is used to construct the phylogenetic tree, which requires the whole chloroplast genome sequence.We performed phylogenetic analysis among six species, including *Iochroma*, *Solanum*, *Hyoscyamus muticus*, *Petunia*, *B. brasiliensis,* and *Convolvulus arvensis*, using *I. batatas*, *I. alba*, *I. aquatica*, and *Convolvulus arvensis* as an outgroup.

## 3. Results

Sequencing reactions can occasionally yield poor-quality reads, often attributed to low coverage or sequencing errors. These low-quality fragments can introduce inaccuracies or noise into the data, potentially resulting in incorrect interpretations of the chloroplast genome structure. Filtering out these fragments helps to maintain the integrity of the genomic data. Genome sequencing of *B. brasiliensis* generated 15.73 M Illumina short-read sequences before filtering.

The chloroplast genome assembly of *B. brasiliensis* is illustrated as a circular structure. This genome, with a length of 169,062 bp, contains a pair of inverted repeat (IR) regions measuring 35,706 bp. Additionally, there is a large single copy (LSC) region measuring 89,315 bp and a small single copy (SSC) region of 8,336 bp, resulting in a total GC content of 37%.

**Figure 2.**
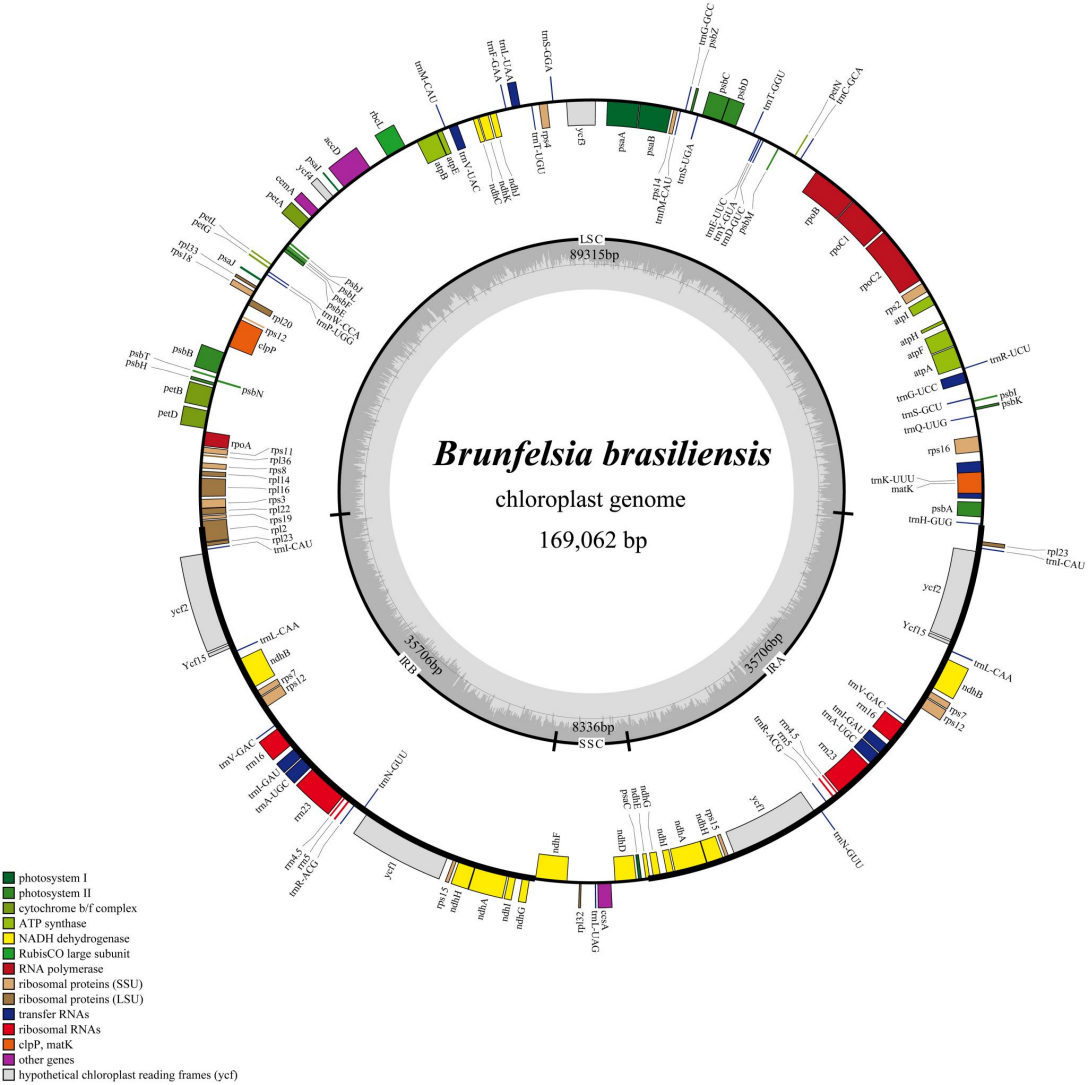
The chloroplast genome map of *B. brasiliensis* illustrates LSC and SSC regions, two IR regions (IRA and IRB), and GC content (light gray). Gene models, including protein-coding genes, tRNA, and rRNA, are represented with various colored boxes along the outer ring. The functional classification of the genes is depicted in the lower-left corner.

**Figure 3.**
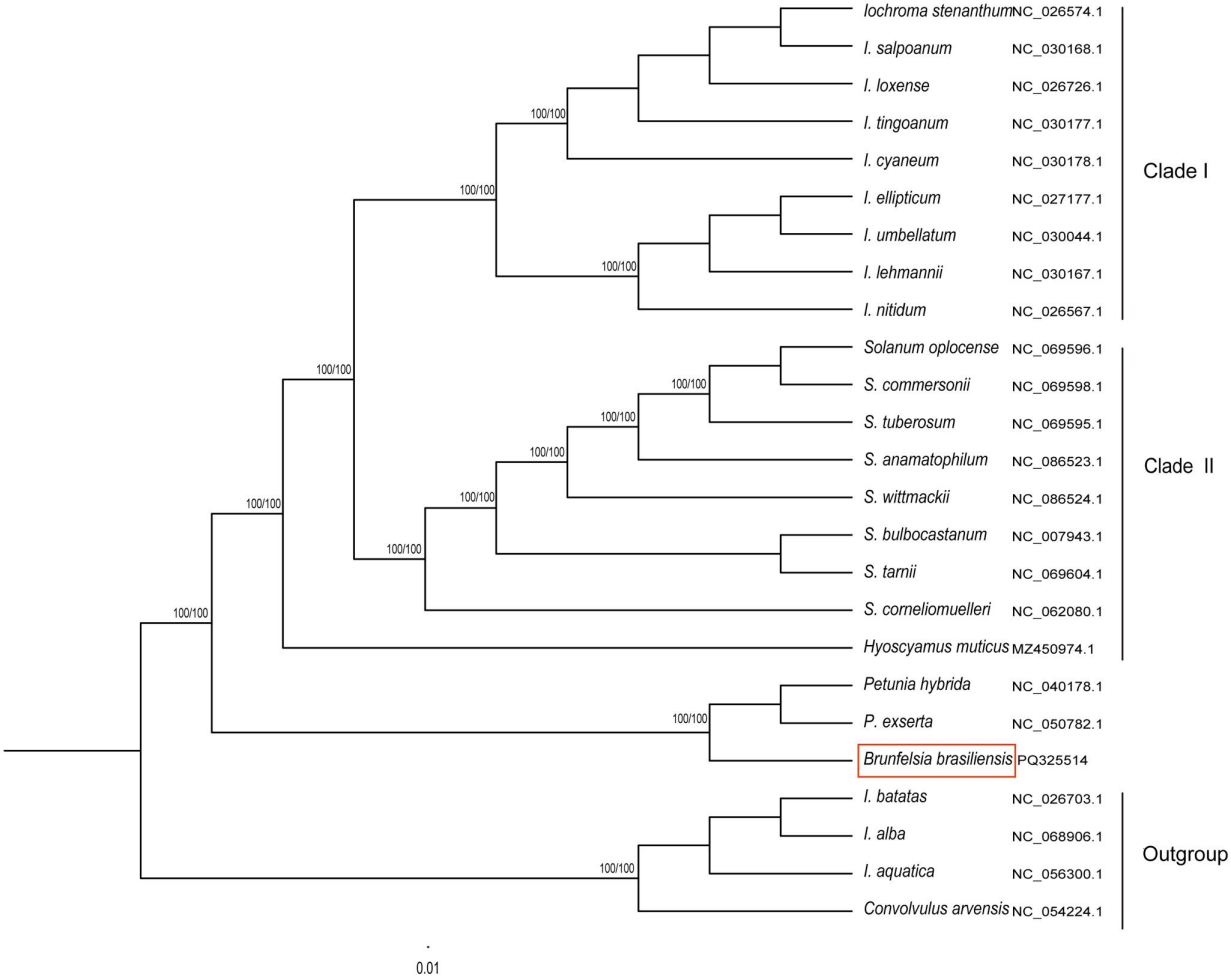
Phylogenetic tree analysis reveals that *B. brasiliensis* shares the closest clustering relationship with *P. hybrida* and *P. exserta*, supported by a 100% bootstrap value. The following sequences were used:. *Iochroma stenanthum* (NC_026574.1), *Iochroma salpoanum* (NC_030168.1), *Iochroma loxense* (NC_ 026726.1), *Iochroma tingoanum* (NC_ 027177.1), *Iochroma cyaneum* (NC_030178.1), *Iochroma ellipticum* (NC_030177.1), *Iochroma umbellatum* (NC_030044.1), *Iochroma lehmannii* (NC_030167.1), *Iochroma nitidum* (NC_026567.1), *Solanum oplocense* (NC_069596.1), *Solanum commersinii* (NC_069598.1), *Solanum tuberosum* (NC_069595.1) (Zhang et al. 2021), *Solanum anamatophilum* (NC_086523.1), *Solanum wittmackii* (NC_086524.1), *Solanum bulbocastanum* (NC_007943.1) (Daniell et al. 2006), *Solanum tarnii* (NC_069604.1), *Solanum corneliomuelleri* (NC_062080.1), *Hyoscyamus muticus* (MZ450974.1) (Magdy et al. 2022), *P. hybrida* (NC_040178.1), *P. exserta* (NC_050782.1) (Sun et al. 2021), *B. brasiliensis* (PQ325514), *Ipomoea batatas* (NC_026703.1) (Zou et al. 2021), *Ipomoea alba* (NC_068906.1) (Sudmoon et al. 2024), *Ipomoea aquatica* (NC_056300.1), and *Convolvulus arvensis* (NC_054224.1) (Wang et al. 2021). *Convolvulaceae Ipomoea* and *Convolvulaceae Convolvulus* were used as outgroup taxa. The plot scale represents a phylogenetic distance of 0.01 nucleotide substitutions per site. The red box indicates *B. brasiliensis.* The species referenced above have already been reported.

The average coverage depth of *B. brasiliensis* cp genome was 384.9x (Supplementary Figure 1). Gene annotation analysis identified 137 genes, including 92 protein-coding genes, 37 tRNA genes, and 8 rRNA genes. Among these, 12 protein-coding genes contain a single intron, while 2 protein-coding genes (*ycf3* and *clpP*) each contain two introns (Figure 2). No chloroplast genome has been reported for any species in the genus *Brunfelsia*. Therefore, we analyzed the evolutionary relationship of *B. brasiliensis* within the *Solanaceae* family. Phylogenetic analysis based on the complete chloroplast genome revealed that *B. brasiliensis* shares the closest clustering relationship with *Petunia hybrida* and *P. exserta* (Supplementary Figure 2), supported by a 100% bootstrap value (Figure 3).

## 4. Discussion and conclusion

Chloroplasts are cellular organelles that possess their own genomes. They are characterized by a simple structure, low molecular weight, moderate evolutionary change, and high levels of conservation (Daniell et al. 2016). With advancements in high-throughput sequencing technologies, chloroplasts are increasingly being used to study the origin, structure, and evolution of organelles (Palmer et al. 1986; Sugiura et al. 1998). Currently, whole chloroplast genome sequences are routinely used in the phylogenetic analysis of plant species. Over 5000 plants have undergone chloroplast genome sequencing, which may be used for species taxonomy, phylogenetics, origin and evolution, and conservation of many valuable traits (Kang et al. 2018; Gao et al. 2020; Sun et al. 2021). Previous studies have revealed that chloroplast genomes are largely conserved and typically exhibit a tetrad shape; however, there are variations in size and base content (Yang et al. 2013; Pogson et al. 2015). The fundamental characteristics of chloroplast genomes provide species-specific genetic information, making them valuable standards for various types of research.

This study assembled the first *B. brasiliensis* chloroplast genome with a length of 169,062 bp and GC content of 37%. The genome structure includes two IRs separated by LSC and SSC regions, similar to those of other plants in the *Solanaceae* family.

*B. brasiliensis* with *Brunfelsia densiflora* Krug et Urb belong to the same family and genus. However, the leaves of *Brunfelsia densifolia* Krug et Urb are quite thin and elongated. The complete chloroplast genome of either species has not been reported. Phylogenetic analysis based on the complete chloroplast genome revealed that *P. hybrida* and *P. exserta* are closely linked to *B. brasiliensis* in evolutionary terms. This first chloroplast genome of the *Brunfelsia* genus plant may offer a valuable genetic resource for further studies on the evolution and diversity within the *Solanaceae* family and *Brunfelsia* species. Furthermore, this *B. brasiliensis* resource may contribute to research on the molecular mechanism underlying color transitions and the biosynthesis of chloroplast-related fragrance components.

In conclusion, this study enriched the chloroplast genome resources for *Solanaceae* family and *Brunfelsia* genus. These findings can foster further genetic research, particularly in the conservation and exploitation of *B. brasiliensis*. As sequencing technologies advance rapidly, the increasing availability of genomic resources will likely uncover detailed phylogenetic relationships within the *Solanaceae* family.

## Supplementary Material

Supplemental Material

## Data Availability

The sequenced data supporting the findings of this study are openly available in NCBI (PQ325514) https://www.ncbi.nlm.nih.gov/search/all/?term=PQ325514, The associated BioProject, SRA, and Bio-Sample numbers are PRJNA1181625, SRR31210101, and SAMN44566118, respectively.
