# Supplementary material for: Characterization of the complete chloroplast genome of Brunfelsia brasiliensis (Spreng.) L.B.Sm. & Downs
Source: Mitochondrial DNA B Resour. 2025 Apr 17;10(5):392–6. doi: 10.1080/23802359.2025.2492096 (PMC12006934; doi:10.1080/23802359.2025.2492096)
Supplement: Supplemental Material [file TMDN_A_2492096_SM5717.docx]

**
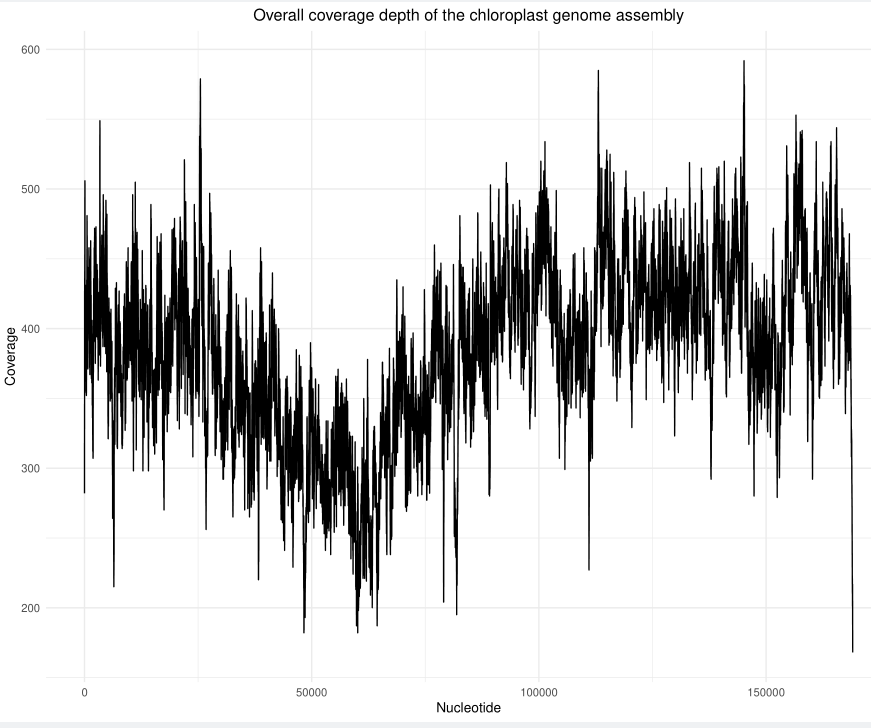
**

Supplementary Figure 1. The average coverage depth of the chloroplast genome of *B. brasiliensis.*


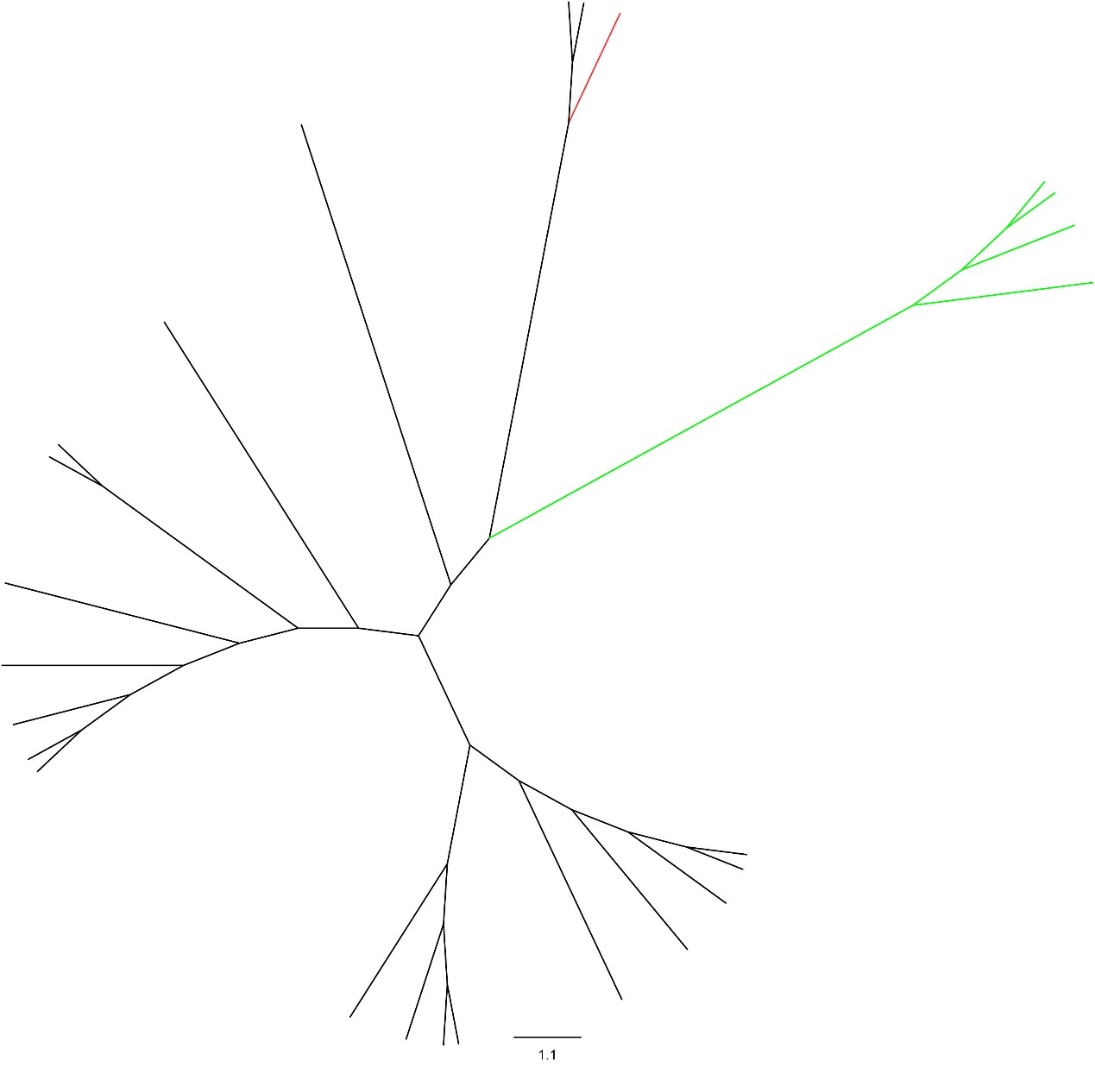


Supplementary Figure 2. Phylogenetic diagram of the chloroplast genome of *B. brasiliensis.* The red line represents the target species, while the green line represents the outgroup.
